# Increased intracellular Cl^-^ concentration improves airway epithelial migration by activating the RhoA/ROCK Pathway

**DOI:** 10.7150/thno.46002

**Published:** 2020-07-09

**Authors:** Wenjie Huang, Meiling Tan, Yue Wang, Lei Liu, Yan Pan, Jingjing Li, Mingxing Ouyang, Chunjiao Long, Xiangping Qu, Huijun Liu, Chi Liu, Jia Wang, Linhong Deng, Yang Xiang, Xiaoqun Qin

**Affiliations:** 1 School of Basic Medicine, Central South University, Changsha, Hunan 410078, China.; 2 Changzhou Key Laboratory of Respiratory Medical Engineering, Institute of Biomedical Engineering and Health Sciences, Changzhou, Jiangsu 213164, China.; 3 Affiliated Liutie Central Hospital of Guangxi medical university, Liuzhou, Guangxi 545007, China.; 4 School of Nursing, Changzhou University, Changzhou, Jiangsu 213164, China.

**Keywords:** Airway epithelial cells, Intracellular chloride, MQAE, Epithelial migration, Cell stiffness

## Abstract

In the airway, Cl^-^ is the most abundant anion and is critically involved in transepithelial transport. The correlation of the abnormal expression and activation of chloride channels (CLCs), such as cystic fibrosis transmembrane conductance regulators (CFTRs), anoctamin-1, and CLC-2, with cell migration capability suggests a relationship between defective Cl^-^ transport and epithelial wound repair. However, whether a correlation exists between intracellular Cl^-^ and airway wound repair capability has not been explored thus far, and the underlying mechanisms involved in this relationship are not fully defined.

**Methods:** In this work, the alteration of intracellular chloride concentration ([Cl^-^]_i_) was measured by using a chloride-sensitive fluorescent probe (N-[ethoxycarbonylmethyl]-6-methoxyquinolium bromide).

**Results:** We found that clamping with high [Cl^-^]_i_ and 1 h of treatment with the CLC inhibitor CFTR blocker CFTR_inh_-172 and chloride intracellular channel inhibitor IAA94 increased intracellular Cl^-^ concentration ([Cl^-^]_i_) in airway epithelial cells. This effect improved epithelial cell migration. In addition, increased [Cl^-^]_i_ in cells promoted F-actin reorganization, decreased cell stiffness, and improved RhoA activation and LIMK1/2 phosphorylation. Treatment with the ROCK inhibitor of Y-27632 and ROCK1 siRNA significantly attenuated the effects of increased [Cl^-^]_i_ on LIMK1/2 activation and cell migration. In addition, intracellular Ca^2+^ concentration was unaffected by [Cl^-^]_i_ clamping buffers and CFTR_inh_-172 and IAA94.

**Conclusion:** Taken together, these results suggested that Cl^-^ accumulation in airway epithelial cells could activate the RhoA/ROCK/LIMK cascade to induce F-actin reorganization, down-regulate cell stiffness, and improve epithelial migration.

## Introduction

Airway epithelia, the first physical barrier, are vulnerable to injuries inflicted by bacterial virulence factors, allergens and environmental hazards. Defective capability epithelial wound repair due to the dysfunction of airway epithelial cells is involved in numerous respiratory illnesses, including chronic obstructive pulmonary disease, asthma, and cystic fibrosis 1, 2. Interest in the overlooked role of intracellular Cl^-^ concentration ([Cl^-^]_i_) in airway epithelial cells has recently increased when a number of studies highlighted the correlation between the above-mentioned diseases and the dysfunction of Cl^-^ channels, such as Ca^2+^-activated chloride channels (CLCs), cystic fibrosis transmembrane conductance regulators (CFTRs), and voltage-gated CLCs in airway epithelial cells 3-8.

Previous researches have shown that the change in [Cl^-^]_i_ is involved in cell proliferation 9, synaptic transmission 10-12, gene expression 13, and NF-κB-activation-related signaling pathways 14, 15. The [Cl^-^]_i_ of liver cancer cells markedly increases in response to acids and alkalis 16. Lipopolysaccharide (LPS) stimulation increases [Cl^-^]_i_ in airway epithelial cells 15, 17. We also found that the [Cl^-^]_i_ of 16HBE14o- cells markedly increases in response to various airway stimuli, including acids, alkalis, coldness, heat, LPS, and H2O2 (Figure S1). Interestingly, in a starfish oocyte wound resealing model, the plasma membrane begins to exhibit chloride permeability by as early as 1 s following wounding 18. This observation prompted us to speculate that intracellular Cl^-^ might be a driving signal of cells in response to injury. The increase in the [Cl^-^]_i_ of injured cells fuels epithelial repair events by launching a signaling cascade that is associated with wound repair.

Cell migration plays an important role in the wound repair of airway epithelial cells. The dynamic polymerization and depolymerization of the filamentous actin (F-actin) cytoskeleton, as well as the remodeling of the mechanical performances of epithelial cells, are involved in multiple stages of cell migration 19, 20. Among several possible control elements of cytophysical properties, the Rho family of GTPases and downstream signals appear to play the most prominent role in modulating the biochemical pathways of cell migration 21-23. Although [Ca^2+^]_i_ is known to be a prominent regulator that can exert multiple effects on the structure and dynamics of the actin cytoskeleton 24, 25, whether [Cl^-^]_i_ is involved this response remains poorly defined.

In this work, we found the increased [Cl^-^]_i_ of 16HBE14o- cells improved epithelial migration, promoted F-actin reorganization, and decreased cell stiffness. High [Cl^-^]_i_ activated RhoA and increased LIMK1/2 phosphorylation without changing intracellular Ca^2+^ concentration ([Ca^2+^]_i_) in 16HBE14o- cells, indicating that [Cl^-^]_i_ could play a vital role in initiating wound repair events and thus promote the biological and mechanical function renewal of 16HBE14o- cells.

## Results

### Double ionophore strategy artificially increased the [Cl^-^]_i_ of 16HBE14o- cells

We used the double ionophore strategy described by Verkman et al. to artificially increase the [Cl^-^]_i_ of 16HBE14o- cells 13, 26, 27 to study the role of Cl^-^ concentration in wound repair. This method enables changes in [Cl^-^]_i_ concentration by changing extracellular concentrations. We clamped the [Cl^-^]_i_ of 16HBE14o- cells at 0 mM to 130 mM. Meanwhile, we used the fluorescence probe MQAE, a low-toxicity indicator of dynamic Cl^-^ concentration in cells, in combination with laser scanning confocal fluorescence microscopy to measure changes in [Cl^-^]_i_. As shown in Figure S2, when [Cl^-^]_i_ was increased from 0 mM to 130 mM, the MQAE fluorescence signal in 16HBE14o- cells was quenched by Cl^-^, and a linear correlation existed between [Cl^-^]_i_ and the changes in MQAE fluorescence. Then, the data were fitted to the Stern-Volmer equation (F_0_/F - 1 = K_SV_[Cl^-^]) with the intracellular quenching constant K_SV_ of 22.71 ± 1.53 M^-1^. In this equation, F_0_ indicates the mean fluorescence level in the high-KNO_3_ clamping buffer (without chloride), and the mean fluorescence at different clamping chloride concentrations is defined as F.

### Increased [Cl^-^]_i_ in 16HBE14o- cells enhanced cell repair activity

**Figure 1:**
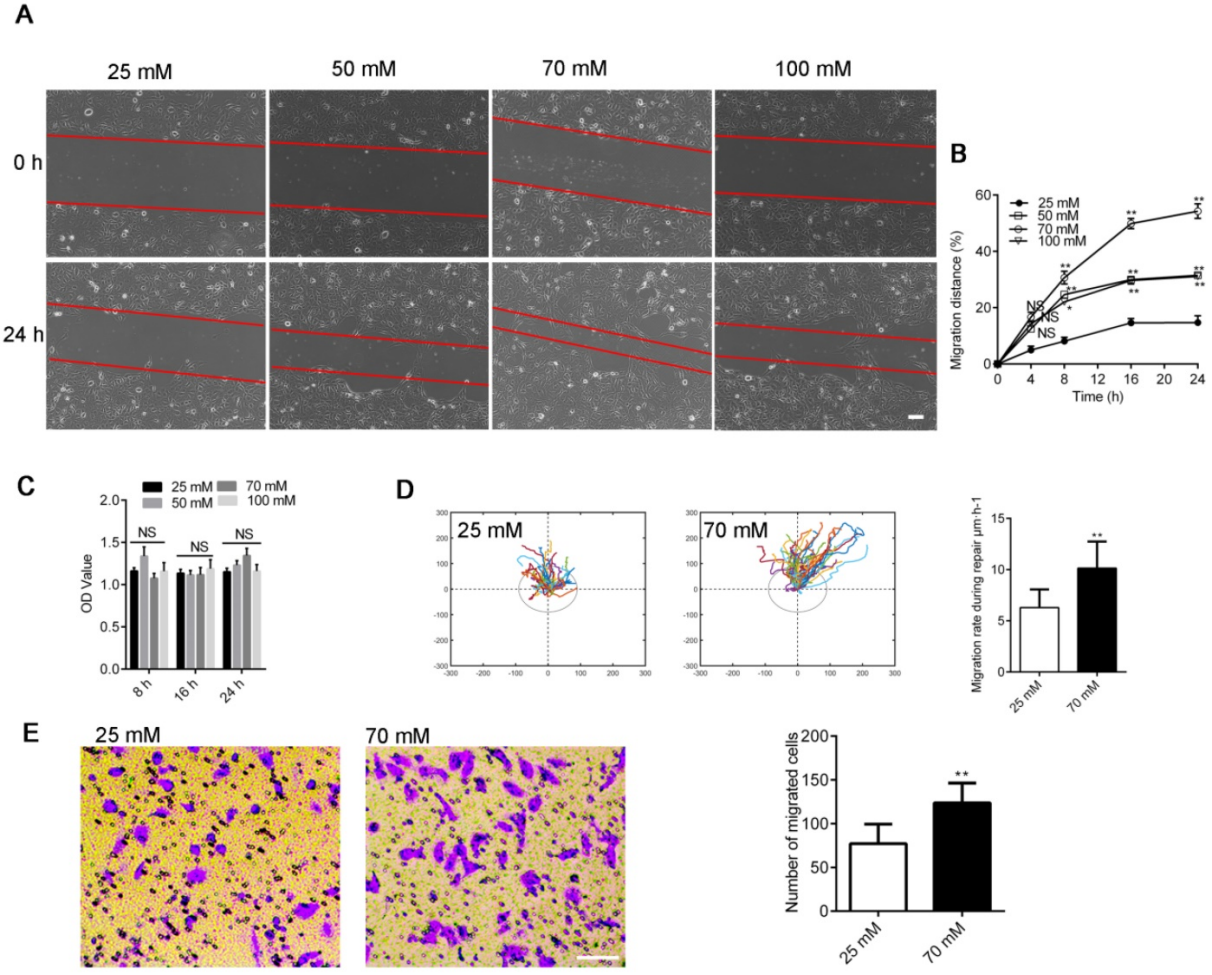
** Effects of clamping [Cl^-^]_i_ at high levels on 16HBE14o- cell wound healing. A)** Cell images taken at 0 and 24 h after injury (Scale bars: 200 µm). **B)** Standardized migration distances at 0, 4, 8, 16 and 24 h after injury. (n = 3 independent experiments, *P < 0.05; **P < 0.01; nonsignificant [NS]). **C)** Proliferation of 16HBE14o- cells was estimated through MTT over different time points (8, 16 and 24 h; n = 3 independent experiments; nonsignificant [NS]). **D)** Mean cell migration rates were calculated from single-cell tracking at the wound edge (45 cells at the wound edge) over a 24 h period after injury (n = 3 independent experiments; **P < 0.01). **E)** Transwell assay following clamping [Cl^-^]_i_ of 16HBE14o- cells at 25 or 70 mM for 1 h. The number of migrated cells was compared between groups (n = 3 independent experiments, **P < 0.01 versus 25 mM, scale bar, 100 µm). Data are presented as mean ± SD.

We assayed wound healing after mechanical injury 28 to investigate repair activities in 16HBE14o- cells to investigate the influence of increased [Cl^-^]_i_ on airway wound repair. [Cl^-^]_i_ was clamped at the concentrations of 25 (resting [Cl^-^]_i_), 50, 70, and 100 mM. As shown in Figure 1A-B, the artificially clamped high levels of [Cl^-^]_i_ markedly promoted wound repair in 16HBE14o- cells in a concentration-dependent manner. The maximum repair rate was shown by the 70 mM [Cl^-^]_i_ group. Furthermore, we tracked and evaluated the migratory capability of different cells in each group by randomly selecting one cell at the wound margin and recording its migration through video-microscopy 6. The mean migration rate of cells in the 70 mM group was significantly faster than that of cells in the group of 25 mM (Figure 1D), and the number of migrating cells was markedly increased in the 70 mM group than in the 25 mM group (Figure 1E). We tested the proliferation of 16HBE14o- cells via the MTT assay to determine if cell proliferation was involved in this process 29. As shown by Figure 1C, the proliferation of 16HBE14o- cells was slightly enhanced although proliferation affected wound repair in response to [Cl^-^]_i_.

### Accumulation of Cl^-^ in 16HBE14o- cells induced by CFTR channels and chloride intracellular channel inhibitors promoted wound repair

**Figure 2:**
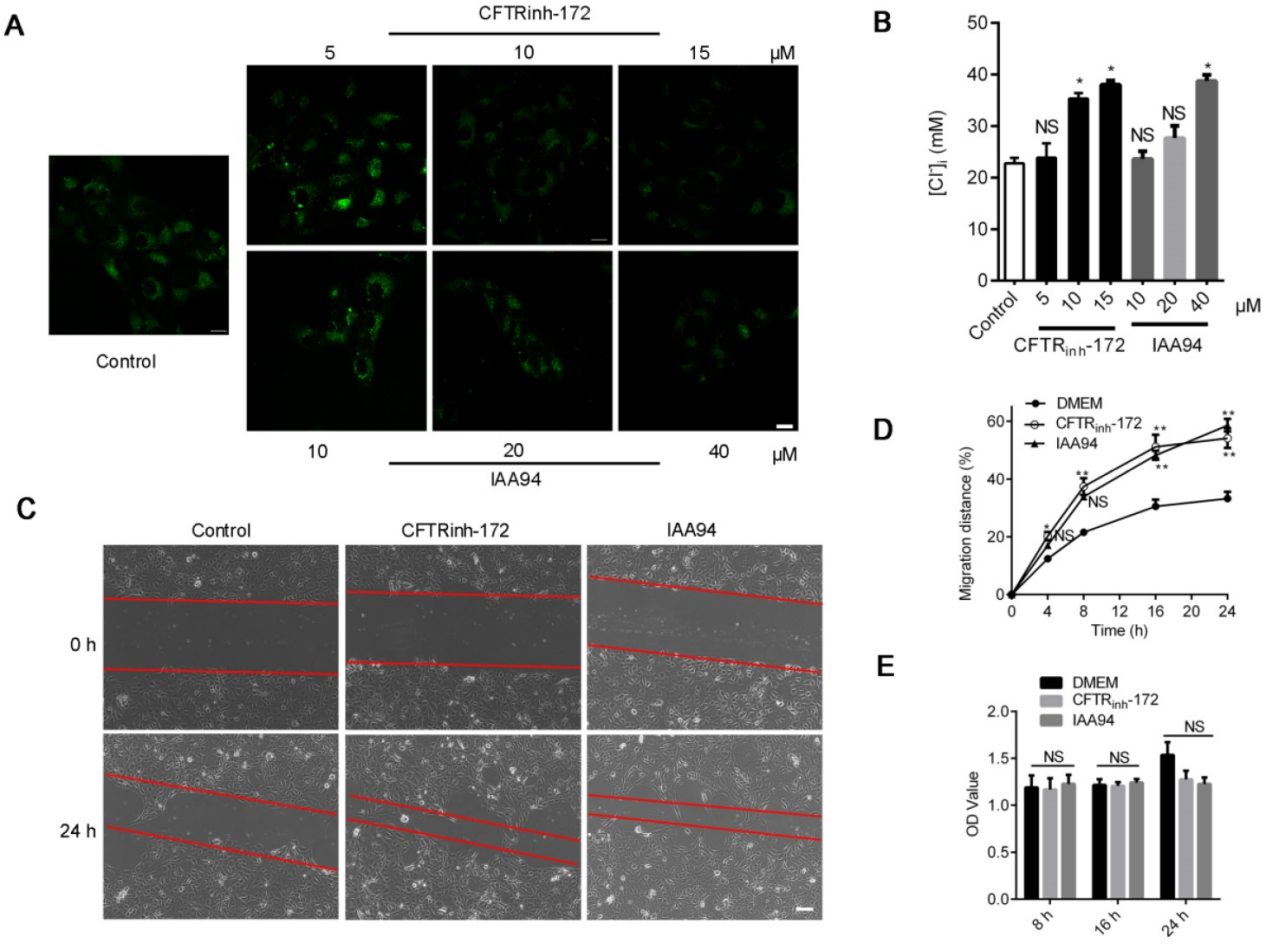
** Effects of CLIC-inhibitor-induced high [Cl^-^]_i_ on the wound-healing capacity of 16HBE14o- cells. A)** Confocal fluorescent images of living 16HBE14o- cells stained with MQAE followed by treatment with CFTR_inh_-172 (5, 10, and 15 µM) and IAA94 (10, 20, and 40 µM) for 1 h (n = 100-180 cells for each group; scale bars: 20 µm). **B)** [Cl^-^]_i_ of 16HBE14o- cells was calculated in accordance with the Stern-Volmer plot. **C, D)** Standardized migration distances were measured at 0, 4, 8, 16 and 24 h after cells were treated with the CFTR blocker CFTR_inh_-172 (10 µM) or the CLIC inhibitor IAA94 (40 µM) for 1 h (n = 3 independent experiments, *P < 0.05; **P < 0.01; nonsignificant [NS]). **E)** Proliferation of 16HBE14o- cells at 8, 16 and 24 h of repair was evaluated via MTT assay (n = 3 independent experiments; nonsignificant [NS]). Data are presented as mean ± SD.

We also induced Cl^-^ accumulation in 16HBE14o- cells through treatment with the CFTR blocker CFTR_inh_-172 (1, 10, and 15 μM) 26 and chloride intracellular channel (CLIC) inhibitor IAA94 (10, 20, and 40 μM) for 1 h 30. As shown in Figure 2A, Cl^-^ accumulated in accordance with the increase in the concentrations of both inhibitors. The Stern-Volmer equation (Figure S2) showed that the [Cl^-^]_i_ of 16HBE14o- cells drastically increased from the baseline value of 22.74 ± 0.83 mM to 35.36 ± 1.29 (CFTR_inh_-172, 10 μM) and 38.74 ± 1.41 mM (IAA94, 40 μM) (Figure 2B). Moreover, as depicted in Figure 2C and D, we found that the high level of [Cl^-^]_i_ induced by CFTR_inh_-172 or IAA94 in 16HBE14o- cells elicited a significant increase in migration rate but only slightly affected cell proliferation (Figure 2E).

### Increased [Cl^-^]_i_ of 16HBE14o- cells promoted cytoskeletal reorganization

**Figure 3:**
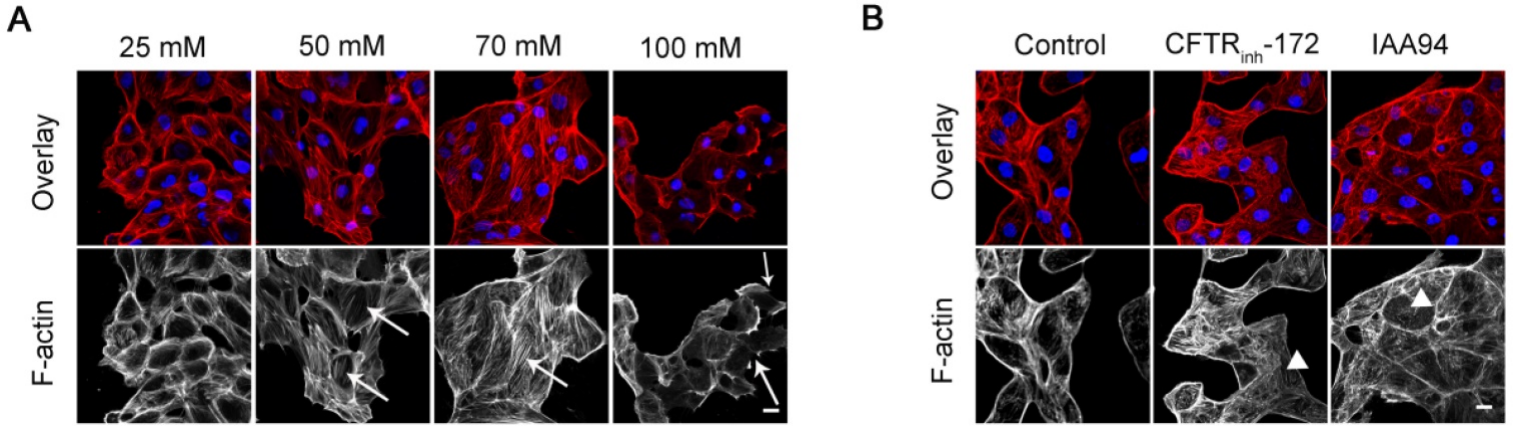
** Effect of increased [Cl^-^]_i_ on the F-actin filaments of 16HBE14o- cells. A)** Confocal images of F-actin filaments in 16HBE14o- cells after incubation with buffers with various chloride concentrations for 1 h. **B)** Confocal images of F-actin filaments in 16HBE14o- cells after treatment with CFTR_inh_-172 (10 µM) and IAA94 (40 µM) for 1 h (n = 3 independent experiments, F-actin, red; nucleus, blue). Scale bars: 20 µm.

Cell migration requires dramatic changes in cell shape. To a large degree, the dynamic remodeling of F-actin is linked to the events of morphological changes and physical forces that occur during migration 31. Typically, 16HBE14o- cells showed highly concentrated F-actin structures around cell peripheries as depicted by Figure 3A and B. The high levels of [Cl^-^]_i_ induced by the double ionophore technique and treatment with CFTR_inh_-172 (10 μM) and IAA94 (40 μM) promoted F-actin reorganization in 16HBE14o- cells. Compared with those of the control cells, the peripheral F-actin fibers of treated epithelial cells were disassembled, and the amounts of threadlike stress fibers were markedly increased throughout the cell body (Figure 3A, arrows; 3B, triangles). The development of perinuclear thick stress fibers is closely associated with elongation capability and contractility 32. The reorganization of F-actin fibers owing to the high level of [Cl^-^]_i_ in epithelial cells suggested an increased potential for migration. However, we also observed numerous discontinuous punctuate peripheral F-actin structures in 16HBE14o- cells treated with 100 mM [Cl^-^]_i_ (Figure 3A, arrows). This result might be in agreement with the finding in Figure 2A, which shows that the migration rate of cells in this group was lower than that of cells in the 70 mM [Cl^-^]_i_ group.

### Increased [Cl^-^]_i_ of 16HBE14o- cells decreased cell stiffness

**Figure 4:**
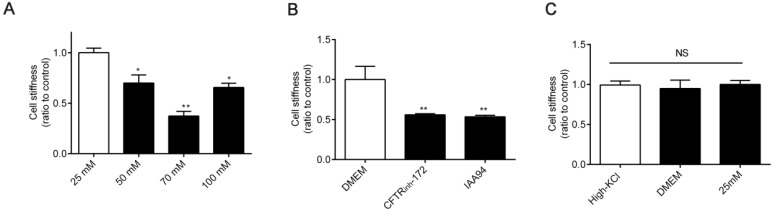
** Effect of increased [Cl^-^]_i_ on cellular mechanical properties. A-B)** Normalized stiffness (G'/G_0_^'^) of 16HBE14o- cells after treatment with buffers with various chloride concentrations and 1 h of treatment with CFTR_inh_-172 (10 µM) and IAA94 (40 µM). **C)** Stiffness of 16HBE14o- cells treated with high-KCl buffer, DMEM, and buffer with 25 mM [Cl^-^]_i_ (resting [Cl^-^]_i_), for 1 h (n = 3 independent experiments, *P < 0.05; **P < 0.01 versus 25 mM). Data are presented as mean ± SD.

The mechanical rigidity of cells is conferred by the cytoskeleton, which forms an internal polymer network that extends throughout the cell body. Therefore, dynamic cytoskeleton remodeling modulates cell stiffness 33, 34. Thus, we next detected cell stiffness by using OMTC 35. As shown in Figure 4A-B, the stiffness of 16HBE14o- cells decreased under treatment with high [Cl^-^]_i_ and 1 h of treatment with CFTR_inh_-172 (10 μM) and IAA94 (40 μM). Cell stiffness did not differ among cells under treatment with DMEM, high-KCl buffer (without ionophores), and 25 mM [Cl^-^]_i_ (resting [Cl^-^]_i_) (Figure 4C).

### Increased [Cl^-^]_i_ in 16HBE14o- cells activated RhoA signaling in 16HBE14o- cells

**Figure 5:**
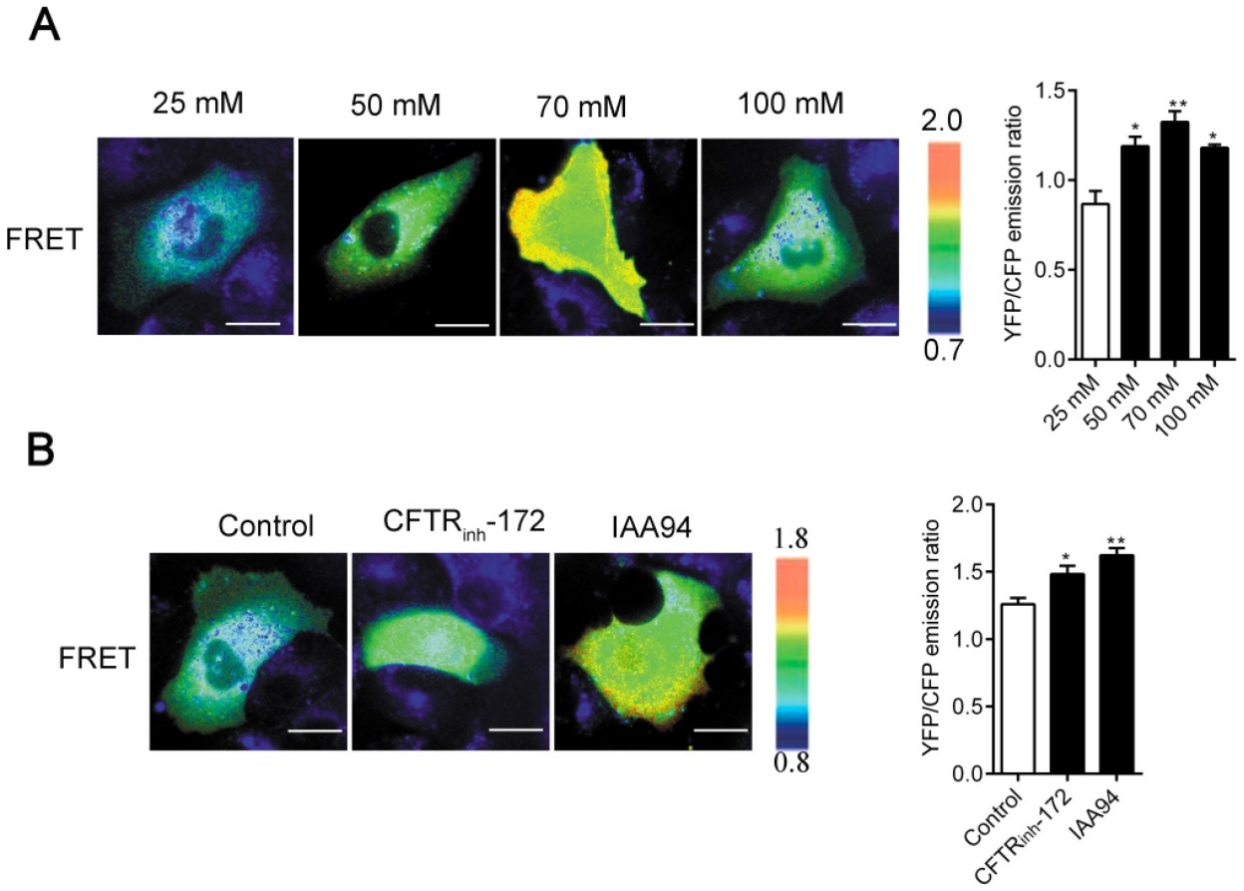
** Increased [Cl^-^]_i_ activated RhoA in 16HBE14o- cells. A)** Images of the emission ratios of YFP/CFP-based RhoA biosensor in 16HBE14o- cells after incubation with buffers with various chloride concentrations. **B)** Images of the emission ratios of the YFP/CFP-based RhoA biosensor in 16HBE14o- cells after 1 h of treatment with CFTR_inh_-172 (10 µM) and IAA94 (40 µM). Cells are shown on the left. The right panels represent the emission ratios of the YFP/CFP-based RhoA biosensors. Emission ratios were measured by taking the mean intensity of a ROI of multiple cells under the same conditions (n = 3 independent experiments; *P < 0.05; **P < 0.01. Scale bars: 20 µm). Data are presented as mean ± SD.

The regulation of cell physical structures is driven by a variety of signaling molecules, among which the most prominent are the Rho family of GTPases. Here, by using the real-time FRET reporters of RhoA activity, we investigated whether increased [Cl^-^]_i_ could activate RhoA signaling in 16HBE14o- cells. As shown in Figure 5A-B, a remarkable increase in RhoA activity was observed in the cells under nigericin and tributyltin treatment (peak induction was observed at 70 mM [Cl^-^]_i_) and those under 1 h of treatment with CFTR_inh_-172 (10 μM) and IAA94 (40 μM). In the control cells, the constitutive activity of RhoA mainly localized at the perinuclear region toward one end of the nuclei. Upon elevated [Cl^-^]_i_ stimulation, a substantial increase in RhoA activity was observed in the whole cytoplasm. These findings suggested that elevated [Cl^-^]_i_ induced the activation of RhoA in airway epithelial cells.

### Elevated [Cl^-^]_i_ of 16HBE14o- cells enhanced the phosphorylation of LIMK1/2 by ROCK1 without changing the [Ca^2+^]_i_ of 16HBE14o- cells

**Figure 6:**
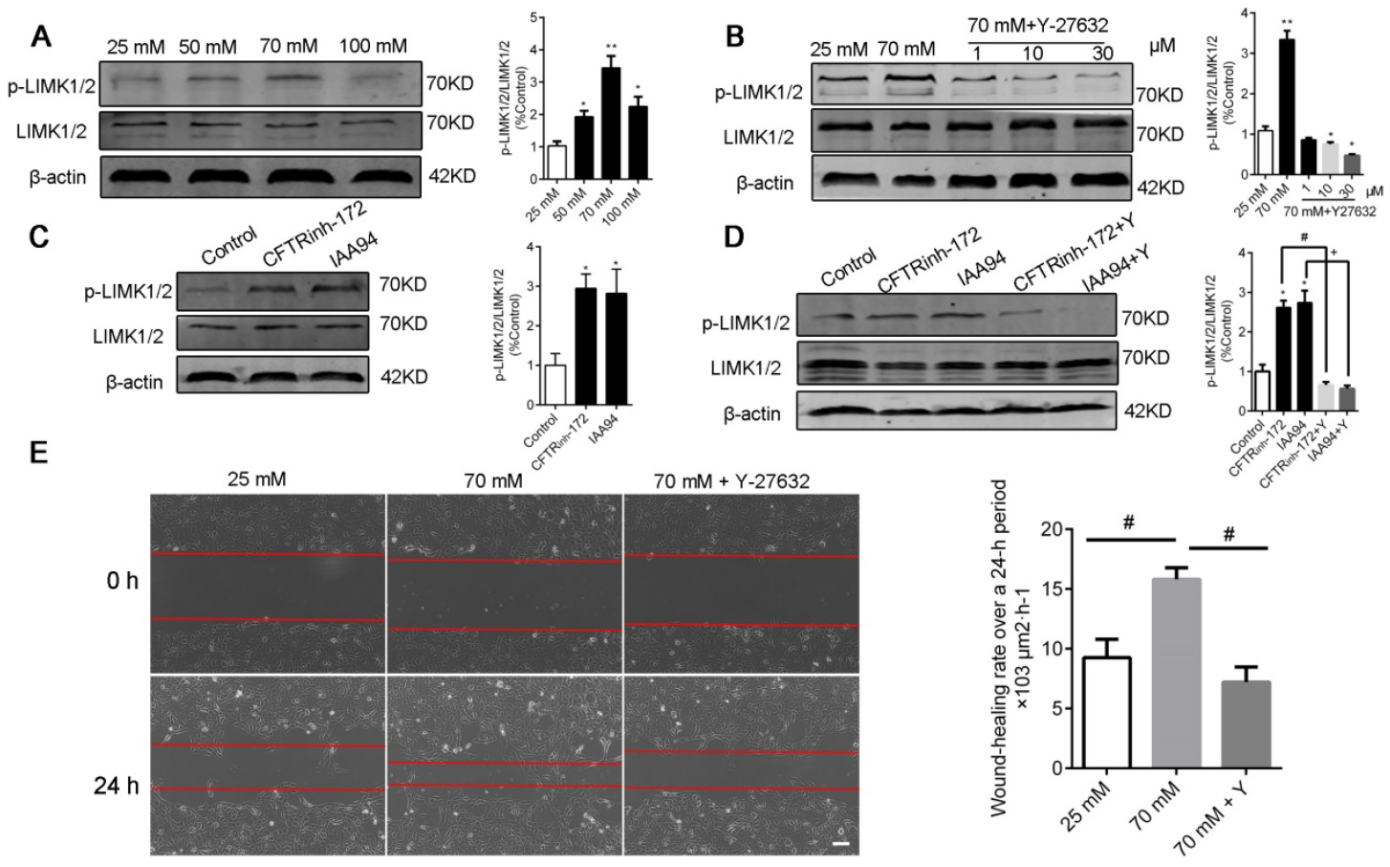
** Inhibition of ROCK abolished the phosphorylation of LIMK1/2 and the improvement in wound healing caused by high [Cl^-^]_i_ in 16HBE14o- cells. A)** Western blot analysis images of LIMK1/2 phosphorylation in 16HBE14o- cells after 1 h of incubation with buffers with various chloride concentrations. **B)** Phosphorylation of LIMK1/2 was measured in 16HBE14o- cells after 1 h of treatment with high [Cl^-^]_i_ in the presence of Y-27632 (1, 10, and 30 µM). **C-D)** Phosphorylation of LIMK1/2 was measured in 16HBE14o- cells after 1 h of treatment with CLICs (CFTR_inh_-172 and IAA94) in the presence of Y-27632 (10 µM). The signal in each lane was quantified by using ImageJ software, and the ratios of p-LIMK1/2/LIMK1/2 to β-actin were determined (n = 3 independent experiments, *P < 0.05; **P < 0.01). **E)** 16HBE14o- cells were wounded, and wound-healing rates were monitored over a 24 h period under high [Cl^-^]_i_ conditions in the presence of Y-27632 (10 µM) for 1 h (n = 3 independent experiments; ^#^*P* < 0.05). Data are presented as mean ± SD.

**Figure 7:**
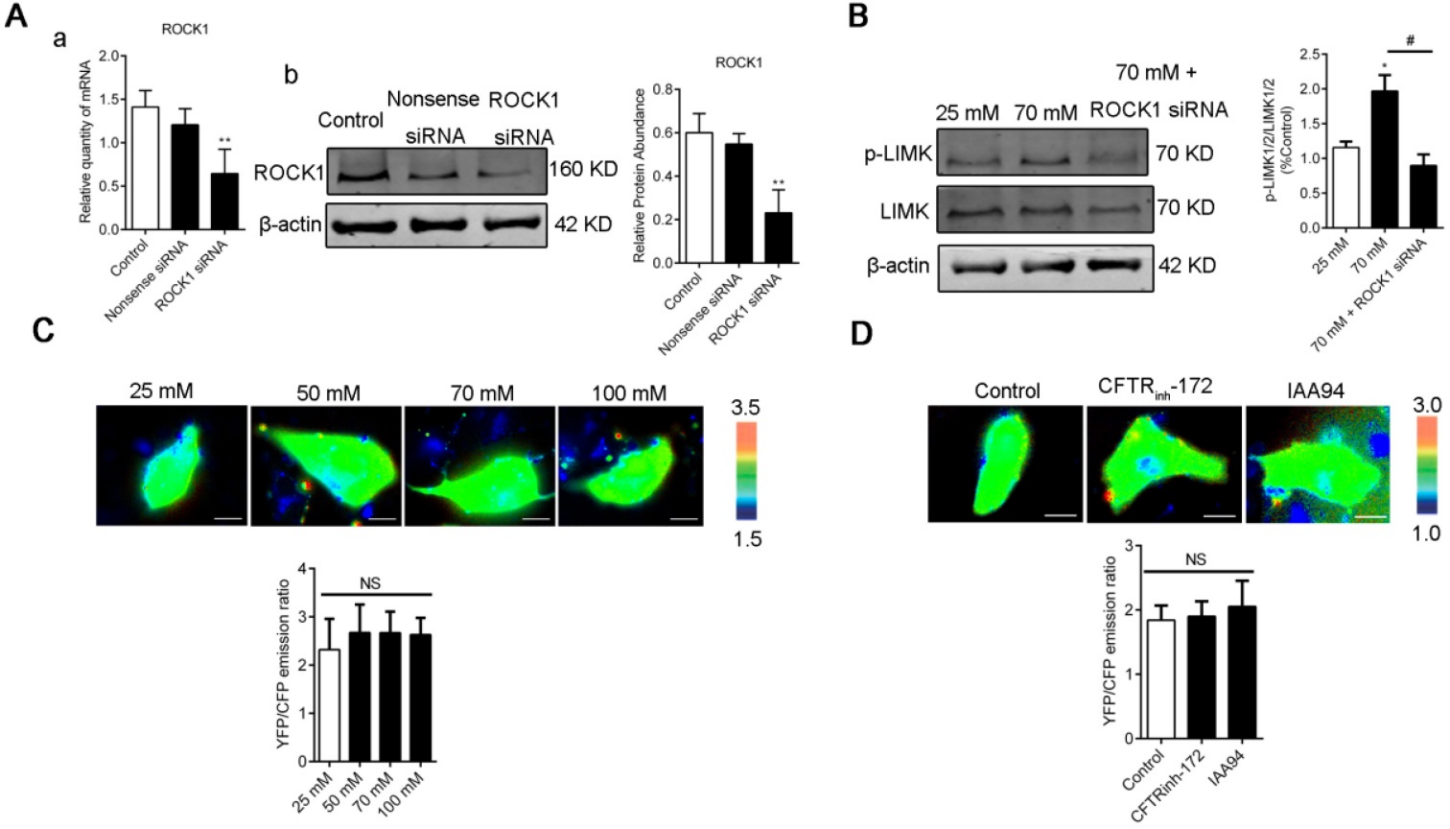
** LIMK1/2 phosphorylation caused by high [Cl^-^]_i_ was regulated by ROCK1 without changing the [Ca^2+^]_i_ of 16HBE14o- cells. A)** ROCK1 RNA and protein levels in 16HBE14o- cells after 48 h of transfection with ROCK1 siRNA were detected through real-time PCR and Western blot analysis accordingly. **B)** LIMK1/2 phosphorylation caused by high [Cl^-^]_i_ in 16HBE14o- cells treated with ROCK1 siRNA was monitored through Western blot analysis. **C-D)** Emission ratio images of the YFP/CFP-based Ca^2+^ biosensor in 16HBE14o- cells after treatment with buffers with various chloride concentrations and 1 h of treatment with CFTR_inh_-172 (10 µM) and IAA94 (40 µM). Cells are shown in the left panel. The right panels represent the emission ratios of the YFP/CFP-based Ca^2+^ biosensor. Emission ratios were measured by taking the mean intensity of a ROI of multiple cells under the same conditions (n = 3 independent experiments; nonsignificant [NS]). Data are presented as mean ± SD.

LIM kinases (LIMK1/2) are serine/threonine kinases that are involved in actin cytoskeletal regulation downstream of the RhoA-ROCK signaling pathway 36. As shown in Figure 6A and C, a high level of [Cl^-^]_i_ increased the phosphorylation of LIMK1/2 in the groups under nigericin and tributyltin treatment and those under 1 h of treatment with CFTR_inh_-172 (10 μM) and IAA94 (40 μM). Under treatment with Y-27632, a specific ROCK inhibitor (Figure 6B and D), or ROCK1 siRNA (Figure 7A-B), the dysregulation of the ROCK pathway significantly abolished the phosphorylation of LIMK1/2 that was induced by a high level of [Cl^-^]_i_. We also observed that ROCK inhibition with Y-27632 (10 μM) decreased the acceleration of wound healing caused by high [Cl^-^]_i_ in 16HBE14o- cells (Figure 6E).

We next examined whether [Ca^2+^]_i_ was affected by [Cl^-^]_i_ clamping buffers or the CLIC inhibitors CFTR_inh_-172 and IAA94. As shown in Figure. 7C-D, [Cl^-^]_i_ increased under treatment with [Cl^-^]_i_ clamping buffers and CLICs without changing the [Ca^2+^]_i_ of 16HBE14o- cells. These data suggested that increased [Cl^-^]_i_ may activate the RhoA/ROCK/LIMK cascade pathway to improve the migration of airway epithelial cells.

## Discussion

Cl^-^ is the most abundant anion in the human body. Previous studies have mainly focused on its functional cellular role in regulating intracellular pH, cell volume, electrical charge balance, salt secretion, and cell reabsorption 1, 37-40. Cl^-^ is also the most important anion that is critically involved in transepithelial transport in the airway. The abnormal expression and activation of CLCs, such as CFTR, ANO1, and CLC-2, are correlated with cell migration capability, suggesting a relationship between defective Cl^-^ transport and epithelial wound repair 6, 8, 41, 42. We previously demonstrated that the expression and function of the CFTR of 16HBE14o- cells (human bronchial epithelial cells) were inhibited under ozone exposure and respiratory syncytial virus infection 43. CFTR failure may delay the wound healing of airway epithelial cells 6. However, whether [Cl^-^]_i_ is implicated in the wound repair of airway epithelial cells remains unclear.

Given that prolonged treatment with nigericin-tributyltin might result in severe cell toxicity, an optimal time for treatment with clamping buffers was used to explore the physical functions of Cl^-^ 13, 15, 27. Here, the [Cl^-^]_i_ of 16HBE14o- cells (25, 50, 70, and 100 mM) was artificially elevated by equalizing [Cl^-^]_i_ and external chloride concentrations for 1 h via the double ionophore strategy and then validated them through ^36^Cl^-^ and 3-O-[^3^H] methylglucose double-label experiments 44. Figure S2 shows the fluorescence changes and the corresponding [Cl^-^]_i_ of 16HBE14o- cells. The K_SV_ was 22.71 ± 1.53 M^-1^, and the mean resting [Cl^-^]_i_ of 16HBE14o- cells in our study was 22.74 ± 0.83 mM. In other tissues, K_SV_ values varied between 3 and 26 M^-1^ 45-48. Kondo and Froemter previously reported cell chloride concentrations between 25 and 30 mM 27. Calculations with the Stern-Volmer equation showed that CFTR and CLIC blockers induced Cl^-^ accumulation inside 16HBE14o- cells by hampering Cl^-^ efflux 49, 50.

Surprisingly, our results demonstrated that elevated [Cl^-^]_i_ caused by CFTR and CLIC blockers improved the migration capability of 16HBE14o- cells (Figure 2). In contrast to our findings, previous results indicate that CFTR mutation or inhibition in cells significantly suppresses Cl^-^ secretion and delays wound healing 6, 7, 15, 51. This discrepancy is likely due to the fact that we only performed 1 h of CLIC treatment to increase [Cl^-^]_i_ instead of stimulating cells for a long time to affect the physiological function of CLCs. Furthermore, we found that cell migration showed the maximal acceleration when [Cl^-^]_i_ was increased to 70 mM and then decreased when [Cl^-^]_i_ was further increased from 70 mM to 100 mM. Interestingly, these results were similar to previous results for the expression of chloride-dependent genes, such as the multifunctional ribosomal protein RPS27, which exhibits biphasic regulation in response to different [Cl^-^]_i_ (25, 75, and 125 mM) 13, 26. During the repair process, cell proliferation in the groups subjected to elevated [Cl^-^]_i_ was not evident in our study. Therefore, the effect of elevated [Cl^-^]_i_ on repair capability observed in this study suggested an intimate relationship between [Cl^-^]_i_ and cell migration.

Cell migration plays a crucial role in the wound repair of airway epithelial cells. The multiple stages of cell migration are closely associated with the dynamic assembly and disassembly of the F-actin cytoskeleton and with the remodeling of the mechanical properties of epithelial cells 52-55. Similar to other ions (for example Ca^2+^ and K^+^), elevated [Cl^-^]_i_ might serve as an intracellular signal and is involved in cell migration 56-58. Our data showed that elevated [Cl^-^]_i_ markedly increased the amount of threadlike stress fibers in the central area of the cells and decreased cytoskeletal stiffness and thus improved cell migration 55. A Transwell assay showed that the number of migrating cells increased when [Cl^-^]_i_ was clamped at a high level, indicating that, in agreement with the reduction in cell stiffness, deformation capacity increased. Notably, the well-organized peripheral F-actin bundles of 16HBE14o- cells showed some disruption when [Cl^-^]_i_ was clamped at 100 mM (Figure 3); this effect was accompanied by an increase in cell stiffness (Figure 4). These results could account for the decreased migration rate of the 100 mM [Cl^-^]_i_ group compared with that of the 70 mM [Cl^-^]_i_ group. The significant inhibition of wound closure in response to the silencing of CFTR expression or the inhibition of CFTR in airway epithelial cells demonstrated in previous studies may be partially ascribed to the function of CFTR as a cytoskeletal complex component that contains ezrin and actin 3, 59.

Cell migration is associated with several signaling cascades and their downstream effectors, including the MAPK cascade, scaffold proteins, and lipid kinase signaling pathways. Nevertheless, the Rho family of GTPases appears to play a prominent role in modulating cell migration 21-23. Moreover, high extracellular NaCl concentration activates GEF-H1, a guanine nucleotide exchange factor (GEF) that directly induces RhoA activation 60. This effect suggests that GEF-H1 may be regulated by [Cl^-^]_i_ and may be implicated in airway epithelial cell migration. However, further experimental verifications are needed. Here, we assessed the activation of Rho GTPases in airway epithelial cells by using a FRET-based method (Figure 5), which accurately reflects cellular RhoA activity. Given that the existence of chloride-dependent genes and multiple Cl^-^ sensing proteins have been documented 13, 15, we hypothesized that the substantial increase in RhoA activity and dispersed distribution in the whole cytoplasm with increased amounts of threadlike stress fibers throughout the cell upon stimulation by [Cl^-^]_i_ elevation might be responsible for cytoskeleton reorganization (Figure 3, indicated by arrows and triangles). Moreover, previous reports have indicated that the phosphorylation of LIM-kinase by ROCK contributes to the Rho-induced reorganization of cellular physical properties in cytoskeletal formation and stiffness 61-63.

Ca^2+^ contributes to RhoA activation and wound repair 64-66. The Cl^-^ conductance stimulated by increased [Ca^2+^]_i_ in epithelial cells exhibits a time-dependent relaxation of the Cl^-^ current 67, 68, suggesting that intracellular Cl^-^ associates with Rho GTPases. Our study showed that elevated [Cl^-^]_i_ activated Rho GTPase and caused LIMK1/2 phosphorylation, which is sensitive to Y-27632 (the Rho-associated kinase ROCK inhibitor) and ROCK1 siRNA, without changing [Ca^2+^]_i_ (Figure 6 and 7). Similar to other studies, our study showed that elevated [Cl^-^]_i_ augmented glucocorticoid inducible protein kinase 1 activity 15 and up-regulated glutaredoxin-related protein 5 13 in a concentration-dependent fashion. These effects plateaued at approximately 70 mM [Cl^-^]_i_. Moreover, in CF cells, the RPS27 gene is positively regulated by 5-75 mM [Cl^-^]_i_ and negatively regulated by 75-125 mM [Cl^-^]_i_ 13. Our study is the first to show that the phospholylation of LIMK1/2 was promoted through biphasic modulation in response to increased [Cl^-^]_i_ and showed maximal promotion under treatment with 70 mM [Cl^-^]_i_. Given that LIM kinases are involved in the reorganization of the actin cytoskeleton 69, we postulated that Cl^-^ may interact with some cytoskeletal regulators. This interaction results in changes in cell stiffness. However, further investigations are needed. Our results were acquired by using cells that were cultured on glass supports. These cells cannot fully reflect the physiological characteristics of polarized epithelial cells. Nevertheless, our results will provide some basic principles related to the relationship among [Cl^-^]_i_, the actin cytoskeleton, and cell movement.

## Conclusion

We investigated the role of intracellular Cl^-^ in the wound repair of bronchial epithelial cells. The RhoA/ROCK/LIMK pathway was activated by elevated [Cl^-^]_i_ to promote the migration of airway epithelial cells. This effect was evidenced by increased RhoA activity, augmented LIMK1/2 phosphorylation, elicited F-actin stress-fiber reorganization, and decreased cellular stiffness. In addition, the ROCK inhibitor Y-27632 and ROCK1 siRNA abolished downstream effects without changing [Ca^2+^]_i_. Our results demonstrated that intracellular Cl^-^ might exert a modulatory effect on the cellular physical properties and functions of airway epithelial cells, thus providing new insight into the pathophysiological function of Cl^-^.

## Materials and Methods

### Cell culture

16HBE14o- cells (an *in vitro* cultured engineered human bronchial epithelial cell line) were obtained from Professor Dieter Gruenert, University of California San Francisco 70, 71. The cells were cultured in high-glucose Dulbecco's modified Eagle's medium (DMEM) containing 10% fetal bovine serum (FBS), 100 U/ml streptomycin, and 100 U/ml penicillin in a humidified air atmosphere containing 5% CO_2_ at 37 °C. Cell culture reagents were purchased from Gibco (Invitrogen, Grand Island, NY, USA). Before the assays, 16HBE14o- cells were starved (no serum) for 12 h. Inhibitors, such as R(+)-IAA94 (40 μM 30; Sigma-Aldrich, St Louis, USA), CFTR_inh_-172 (10 μM 15; Sigma-Aldrich), and Y-27632 (10 μM, Sigma-Aldrich, St Louis, USA) were added to the cells for 1 h.

### Measurement of the Stern-Volmer constant for N-(ethoxycarbonylmethyl)-6-methoxyquinolium bromide quenching by Cl^-^

The double ionophore strategy was used with some modifications as previously described 13, 27 to define the relationship between the fluoescence intensity of the quinolinium-salt-based halide-sensitive fluorescence probe N-(ethoxycarbonylmethyl)-6-methoxyquinolium bromide (MQAE; ab145418, Abcam) and [Cl^-^]_i_ 72. Briefly, 16HBE14o- cells were cultured on glass-bottomed cell culture dishes (801002, NEST, China). The cells were subsequently loaded with 5 mM MQAE in the dark for 1 h at 37 °C, washed twice to remove the unbound probe, and then clamped with different [Cl^-^]_i_ for 1 h. Solutions with different [Cl^-^]_i_ were prepared by mixing two high-K^+^ buffers (high KO_3_ buffer [in mM]: KNO_3_ [140], CaCl_2_ [1.3], NaH_2_PO_4_ [3.7], KH_2_PO_4_ [0.4], NaHCO_3_ [4.2], MgSO_4_ [0.7], HEPES 10, D-glucose [5.5] [pH = 7.4]; High-KCl buffer [in mM]: KCl [140], CaCl_2_ [1.3], NaH_2_PO_4_ [3.7], KH_2_PO_4_ [0.4], NaHCO_3_ [4.2], MgSO_4_ [0.7], HEPES 10, and D-glucose [5.5] [pH = 7.4]) containing the Cl^-^/OH^-^ exchanger tributyltin (10 μM) and K^+^/H^+^ exchanger nigericin (5 μM) to equilibrate extracellular and intracellular pH and Cl^-^. As previously described, 15 min of tributyltin/nigericin treatment was sufficient to equilibrate [Cl^-^]_e_ and [Cl^-^]_i_, and osmolarity was in the normal range (293-302 mOsm/Kg) 27. Fluorescence intensity was recorded by using a laser scanning confocal microscope (Zeiss LSM710, Carl Zeiss, Jena, Germany). Fluorescence images of MQAE were acquired at an excitation wavelength of λ = 350 nm. Then, in accordance with the Stern-Volmer plot (F_0_/F = 1 + K_Cl_ [Cl^-^]), the calibration curve was acquired by fitting fluorescence intensity to the corresponding [Cl^-^]_i_. In this equation, F_0_ represents the mean fluorescence level of the Cl^-^-free clamping solution (high-KNO_3_ buffer), and the mean fluorescence intensity at different clamping [Cl^-^]_i_ is defined as F. The Stern-Volmer constant K_Cl_ (M^-1^) is the reciprocal of a linear regression fit 73.

### Cell stiffness measurement

The stiffness of 16HBE14o- cells was measured through optical magnetic twisting cytometry (OMTC). The details of this method have been previously described 74. Briefly, cells were incubated with RGD-coated magnetic ferrimagnetic beads (4.5 μm in diameter) at 37 °C for 20 min. The beads were fabricated in Dr. Jeffery Fredberg's lab at the Harvard School of Public Health. Subsequently, the cells were washed with PBS for three times to remove unbound beads. The cells were placed on a microscope stage with magnetization and twisting coils (OMTC-1D, EOL, Switzerland). The beads were rotated and displaced by a homogeneous magnetic twisting field. Then, images were acquired during a twisting cycle by using a Cell Observer System (Zeiss, Göttingen, Germany). The stiffness of F-actin (G′) was calculated by using the ratio of the applied magnetic torque to the measured lateral bead displacement. The measurement of G′ was repeated 12 times under each experimental condition by using Matbin. We defined G′_0_ as the baseline cellular stiffness, and G′ was normalized to G′_0_ in each experiment to compare stiffness values among different experimental batches and groups.

### Immunofluorescence assay

Cells were washed three times with PBS and then fixed with 4% paraformaldehyde for 15 min at room temperature. Cells were then rinsed three times with PBS (2 min each time) and subsequently permeabilized with 0.3% Triton X-100 in PBS for 5-6 min. Cells on slides were blocked with 10% BSA in PBS for 30 min at 37 °C. F-actin was stained with phalloidin (teramethylrhodamine isothiocyanate-phalloidin, 1:200 dilution, Yesdrn Biotechnology Co. Ltd, China) at room temperature for 30 min. Nuclei were stained with 4′,6-diamidino-2-phenylindole (Sigma-Aldrich, USA) for 2-3 min. Fluorescence images were taken by using a laser scanning confocal microscope (Zeiss LSM710, Carl Zeiss).

### Wound healing assay

The wound healing assay was conducted with a modified method as previously described 28, 75. Briefly, human airway epithelial cells were grown to 100% confluence and then starved for 12 h. The cells were then clamped with different [Cl^-^]_i_ and treated with the CLC inhibitors CFTR_inh_-172 and IAA94 for 1 h after mechanical injury with a p200 pipette tip. The cells were subsequently washed twice, and culture media were substituted with starvation medium (DMEM without FBS and inhibitors). Wound areas were observed and recorded at 0, 4, 8, 16, and 24 h by using a Cell Observer System (Zeiss, Germany) equipped with a CO_2_ control chamber and temperature. The rate of wound closure after repair was measured by applying ImageJ software (Image-Pro Plus, Version 7.0) and compared with the initial wound area, which was calculated from three representative wound areas and presented in μm^2^·h^-1^. ImageJ software and Matlab (The MathWorks) were used to analyze migration rate and trajectories.

### Transwell assay

Cell migration was measured by using Transwell chambers (Corning, New York, USA) in accordance with previously described 76. Briefly, the cells were clamped with different [Cl^-^]_i_ for 1 h and then plated in Transwell chambers at a density of 2 × 10^4^ in 200 μl of serum-free medium. Next, 800 μl of medium with 10% FBS was added into the lower chambers. After 24 h of incubation at 37 °C, the cells that had migrated to the lower surface were fixed with 4% paraformaldehyde for 15 min and stained with 0.5% crystal violet (Ameresco, LLC, USA) for 6 min. Then, the migrating cells were photographed with an inverted microscope (×200 magnification) and counted by using Image J software.

### Cell proliferation assay

The proliferation of 16HBE14o- cells was evaluated through MTT (3-[4,5-dimethylthiazol-2-yl]-2,5-diphenyl-tetrazolium bromide) assay as previously described 28. Briefly, cells were inoculated into 96-well assay plates at a density of 10^4^ cells per well and then starved for 12 h before stimulation with different [Cl^-^]_i_, CFTR_inh_-172, and IAA94. Then, the cells were incubated with 100 μl of 0.5% MTT solution in each well at 37 °C for 4 h. Subsequently, the supernatants were removed, and dimethyl sulfoxide (AR, Yonghua chemical Technology, China) was added to each well. The mixture was shaken for 10 min to dissolve crystals. Absorbance was acquired by using an automatic microplate reader at 570 nm (Elx100, Thermo Fisher Scientific, Inc, Waltham, MA, USA).

### Fluorescence resonance energy transfer microscopy of 16HBE14o- cells

Biosensors with intramolecular fluorescence resonance energy transfer (FRET) that responded to RhoA activation were used to study RhoA activation in living epithelial cells. The RhoA FRET biosensor used in this work was gifted by Professor Klaus Hahn at University of North Carolina and designed as previously described 77. This biosensor contains a Rho-binding domain (RBD), which specifically binds to GTP·RhoA. This domain is followed by a cyan fluorescent protein (CFP), an unstructured linker of optimized length, a yellow fluorescent protein (YFP), and full-length RhoA. Upon activation by GTP-loading, RBD binds to Rho, changing the relative orientation of the two fluorophores and thus increasing FRET. RhoA activation is approximately proportional to the FRET/CFP emission ratio due to the attachment of fluorescent proteins to one another. Similarly, intracellular Ca^2+^ was detected by using a fluorescent Ca^2+^ biosensor that is based on green fluorescent proteins and calmodulin 78, 79. The binding of Ca^2+^ causes calmodulin wrapping, thus increasing the FRET between the flanking GFPs.

After transfection with RhoA and Ca^2+^ biosensors for 36-48 h, 16HBE14o- cells were seeded on fibronectin-coated 15 mm glass-bottomed cell culture dishes (801002, NEST, China) and then starved in 0.5% FBS for 12 h before stimulation. Cell images were collected with a Cell Observer System (Zeiss), which was equipped with a CO_2_ control chamber and temperature. The following filters were used to acquire images (excitation; dichroic; emission) CFP (424/24 nm; 455; 460/40 nm), and YFP (426/20 nm; 455; 520/30 nm). The emission ratios of YFP/CFP were computed and generated by using Excel (Microsoft) and MetaFluor software to reflect RhoA activation and Ca^2+^ signals throughout the live cell.

### Western blot analysis

Western blot analysis was conducted with a modified method as previously described 76, 80. Briefly, the total protein of the cells was collected with RIPA Lysis Buffer (Thermo Scientific, USA) containing 1% phenylmethanesulfonyl fluoride on ice. After the detection of protein concentration by using a BCA Kit (Takara, Tokyo, Japan, T9300A), the cell lysates (50 μg of protein) were subjected to 10% sodium dodecyl sulfate-polyacrylamide gel electrophoresis and subsequently transferred onto a PVDF membrane that was blocked with 5% bovine serum albumin (BSA) and then incubated overnight at 4 °C with the following primary antibodies: β-actin (Sigma-Aldrich, St Louis, MO, USA, A5441), LIMK1/2, and p-LIMK1/2 (1:1000, Affinity, USA). Then, the membrane was washed with TBST (TBS 0.1% Tween-20) three times and subsequently incubated with the following secondary antibodies at room temperature for 1 h: IRDye800CW antimouse IgG and goat antirabbit IgG diluted 1/5000 from LO-COR, Biosciences (Lincoln, NE, USA). Immunoblots were evaluated by using an Odyssey Imaging System, and β-actin was used as the loading control.

### Silencing of gene expression via small interfering RNA technique

Small interfering RNAs (siRNAs) were transfected into the indicated cells by using Lipofectamine 3000 (Thermo Fisher Scientific) in accordance with standard procedures. The human ROCK1-gene-specific siRNA sequences (designed and synthesized by Ribobio lnc.) were as follows: 5′-GTT CTA TAA TGA CGA ACA A-3′ for siRNA-1, 5′-GCT GCA AGC TAT ATT AGA A-3′ for siRNA-2, and 5′-GTA GAA GAA TGT GCA CAT A-3′ for siRNA-3. The nonsense siRNA sequence 5′-TTC TCC GAA CGT GTC ACG T-3′ was used as the negative control. The efficiency of gene silencing after siRNA transfection was detected through Western blot and real-time PCR analyses.

### Statistical analysis

Statistical analysis was conducted by using GraphPad Prism 5.01 software (Graph-Pad Software, San Diego California, USA). Data were presented as mean values ± SD (1 standard deviation) from a representative experiment. Each experiment was independently carried out at least three times. Student's *t*-test (two-tailed) was used to evaluate the statistical difference between groups. Data for three or more groups were analyzed with one-way analysis of variance followed by Bonferroni for multiple comparisons. *P* < 0.05 was assumed to denote statistical significance.

## Supplementary Material

Supplementary figures.
