# Supplementary material for: Increased intracellular Cl- concentration improves airway epithelial migration by activating the RhoA/ROCK Pathway
Source: Theranostics. 2020 Jul 9;10(19):8528–40. doi: 10.7150/thno.46002 (PMC7392015; doi:10.7150/thno.46002)
Supplement: Supplementary file 1 — Supplementary figures. [file thnov10p8528s1.pdf]

Supplementary figure

Figure S1

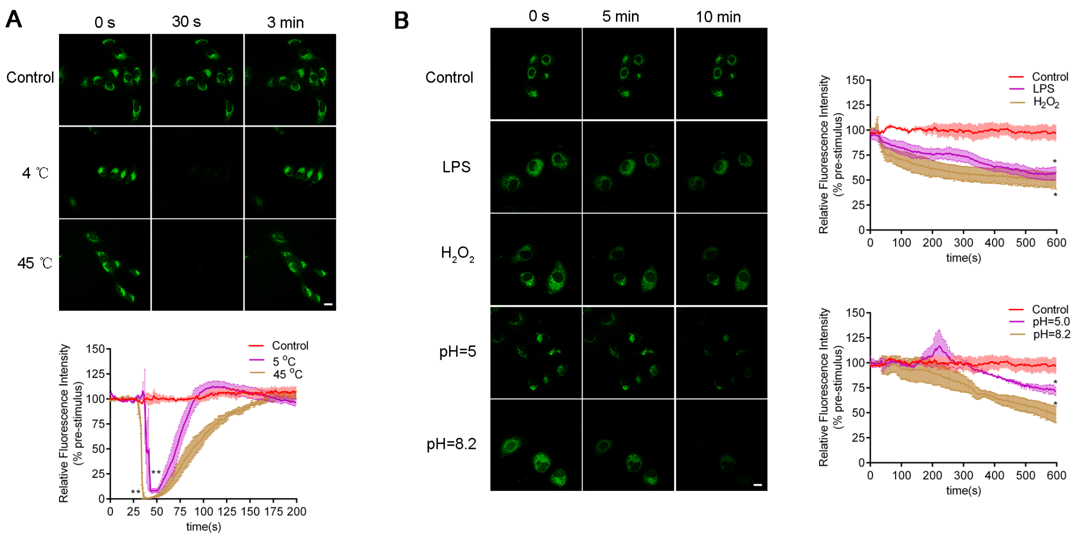

Figure S1. Various stimuli increase intracellular Cl^- levels in 16HBE14o- cells. 16HBE14o- cells were cultured on Glass Bottom Cell Culture Dish (801002, NEST, China). Cells were subsequently loaded with 5 mM MQAE(N-(ethoxycarbonylmethyl)-6-methoxyquinolium bromide; ab145418, Abcam) in the dark for 1 h at 37 ℃ and washed twice to remove the unbound probe. Then, various stimuli including cold (5 ℃), hot (45 ℃), acid (pH=3.6), alkali (pH=8.2), hydrogen peroxide (H_2O_2, 15 mM) and LPS (35 μg/ml) HEPES buffers were added to the culture dish. Confocal microscopy scanning of the process was accomplished within 3-10 min. In each set of experiment, images were taken using the same parameter settings.

A-B) Left panel shows representative confocal microscopy images of 16HBE14o- cells, which were loaded with MQAE (a Cl^- indicator), before and after application of coldness (4℃), hot (45℃), acid (pH=5), alkali (pH=8.2), H_2O_2 (15 mM) and LPS (35 μg/ml) to the basolateral bath. Right panel shows summary plots of relative fluorescence intensity of MQAE indicating the

change levels of intracellular Cl^- (n=3 independent experiments; *P< 0.05; **P<0.01).

Figure S2

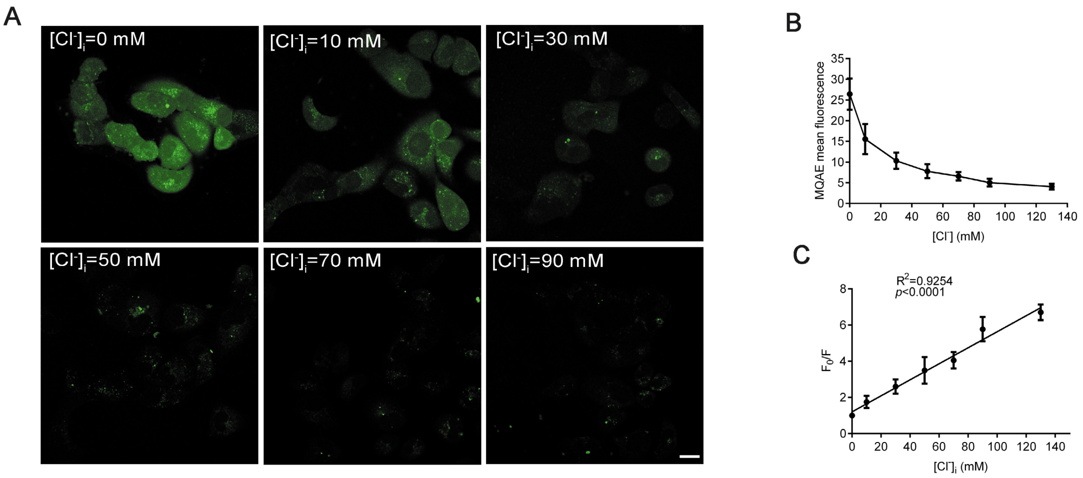

Figure S2. Intracellular quenching of MQAE by chloride ion in 16HBE14o- cells. **A-B)** Correlation between the intracellular chloride concentrations and the mean fluorescence intensities of MQAE were obtained in 16HBE14o- cells (n=100-180 cells for each group; Scale bars: 20 μm). Cells were incubated with buffers at various chloride concentrations (0, 10, 30, 50, 70, 90 and 130 mM) in the presence of the ionophores tributyltin (10 μM) and nigericin (5 μM), for 1 h. **C)** Stern-Volmer plot analysis ($F_0/F-1=K_{Cl^-}$ $[Cl^-]$) for the double ionophore strategy. The regression line gives the Stern-Volmer constant (K_{sv}; slop of the line) of 22.71 ± 1.53 M^{-1} (n=3 independent experiments). Data are presented as mean ± SD.
